# New ways: the pandemics of science fiction

**DOI:** 10.1098/rsfs.2021.0027

**Published:** 2021-10-12

**Authors:** Glyn Morgan

**Affiliations:** Science Museum, London, UK

**Keywords:** pandemics, science fiction, COVID-19, cultural studies, English literature

## Abstract

In unprecedented times, people have turned to fiction both for comfort and for distraction, but also to try and understand and anticipate what might come next. Sales and rental figures for works of fiction about pandemics and other disease outbreaks surged in 2020, but what can pandemic science fiction tell us about disease? This article surveys the long history of science fiction's engagement with disease and demonstrates the ways in which these narratives, whether in literature or film, have always had more to say about other contemporary cultural concerns than the disease themselves. Nonetheless, the ideas demonstrated in these texts can be seen perpetuating through the science fiction genre, and in our current crisis, we have seen striking similarities between the behaviours of key individuals, and the manner in which certain events have played out. Not because science fiction *predicts* these things, but because it anticipates the social structures which produce them (while at the same time permeating the culture to the extent that they become the touchstones with which the media choose to analyse current events). This paper demonstrates that science fiction can be a valuable tool to communicate widely around a pandemic, while also acting as a creative space in which to anticipate how we may handle similar events in the future.

## 1.

At the COVID-19 pandemic's first peak, amid confusion and conflicted messaging from some news and political sources, English-language audiences flocked to science fiction. Often portrayed as escapism, science fiction fans and critics will be quick to point out that in fact the genre is most interested in reflecting contemporary concerns and anticipating future ways of thinking. Yet, even if we accept this misconception for a moment, readers and viewers were drawn not to strange new worlds in galaxies far, far away, but to narratives of apocalyptic disease and societal disarray. Steven Soderbergh's 2011 film *Contagion* was, by March, the second most streamed and rented film in the Warner Bros. back catalogue, jumping up from number 270 [1]. In print, *The Eyes of Darkness*, the 1981 novel by horror writer Dean Koontz which imagines the catastrophic effects of a new virus ‘Wuhan-400’, experienced an e-book sales surge of more than 3000% in just three weeks [2]. Spikes in sales have been seen in Albert Camus' *The Plague* (1947), Stephen King's *The Stand* (1978) and Emily St John Mandel's *Station Eleven* (2014), among many others. Of course, sales data do not track those who are returning to copies of books they already own or digging out DVDs they have owned for years. Rather than seeking imaginary worlds in which to hide from anything that reminds us of our changed world, it seems many of us are choosing to explore how we have imagined global pandemics in years gone by. Two centuries of science fiction have tracked the cultural and social responses to ideas of illness on a global scale, whether addressing pandemics directly or using viral and bacteriological threats as metaphor, and thus, the genre is a rich repository of ideas against which we can weigh our COVID-19 experiences.

Science fiction is a narrative art form which relies on imaginative and cognitive leaps of extrapolation to create new worlds of fiction distinct in some way (sometimes small, sometimes large) from our own. ‘Such new textual worlds', writes Damien Broderick, ‘are set off from ours chiefly by means of a drastic disruption, an anomalous breach in accepted verities’ [3]. The disruptor might be a new technology such as time travel or cheap interplanetary travel, or an unprecedented event such as alien invasion or all-out nuclear war. Collecting these phenomena together, theorist Darko Suvin, refers to a literary device such as the ‘novum’ or new thing [4]. The irony is that plagues, epidemics and disease are not a new thing, they have been with us throughout human history, even shaped our evolution and can be found globally across all forms of literature [5].^1^ However, what science fiction has done throughout its history is exploit the spectre of disease in service of the genre's other defining feature, what Suvin calls ‘cognitive estrangement’, a process of employing the thought processes and language of science and scholarship to create new worlds [4]. Through this process, authors (and film makers, artists and other producers of science fiction) are able to establish new viewpoints from which to look back on our own world and reflect on the issues most important to them and their contemporaries.

The earliest example of this cognitive estrangement of disease takes us back to the very foundations of the genre with the works of Mary Shelley. Shelley's best-known work, *Frankenstein* (1818), is often championed as the first science fiction novel, ushering in a blending of the Gothic novel, Romanticism and scientific extrapolation that would shape the genre for centuries. However, less well remembered is *The Last Man* (1826), Shelley's portrayal of a global pandemic which causes the collapse of European civilization until our narrator believes himself the last human alive, writing the book as testimony despite there being no one left to read it.

*Frankenstein* is often considered alongside biographical themes of birth and death from Shelley's life, and so too we can see the more morbid elements of Shelley's imagination given full flight in *The Last Man* in the wake of the death of her husband, the Romantic poet Percy Shelley, and his fellow poet and close friend Lord Byron in the years immediately preceding the publication of the novel.^2^ The nineteenth century also saw a spate of cholera epidemics and while the disease had yet to reach Britain when Shelley was writing the book, the epidemic in India killed millions and raged for 7 years until 1824. Closer to home, an outbreak of typhus emerged in Ireland between 1816 and 1819 killing more than 65 000 people [6].

Although *The Last Man* sold moderately well upon publication, it was poorly received in its time with critics writing harshly about the novel's morbidity, although it seems the perceived anti-monarchist politics of the novel were as much cause for offence as anything else. Scientifically, the novel is a product of its day and Shelley's characters refer to the disease (which has attributes of various real diseases including plague, cholera and scarlet fever) in terms of miasma rather than germ theory, with characters praising the winter season as ‘a general and never-failing physician. […] The effects of purifying cold were immediately felt; and the lists of mortality abroad were curtailed each week’ [7]. As germ theory became better understood and captured the popular imagination, science fiction narratives adapted and later in the same century, we see examples such as H. G. Wells' short story ‘The Stolen Bacillus’ (1894) which draws on the scientific contexts of his day, and was no doubt inspired by the actions of French anarchist Martial Bourdin who was killed earlier in 1894 when the chemical explosives he was carrying detonated prematurely at The Royal Observatory, Greenwich. In Wells' story, an anarchist attempts to steal a vial of ‘Asiatic Cholera’ which he plans to introduce to London's water supply [8].^3^ Wells also famously employs a pandemic in humanity's defence in his 1898 novel *The War of the Worlds,* where the invading Martians are not repelled by the might of the British military but instead are ‘slain by the putrefactive and disease bacteria against which their systems were unprepared; […] slain, after all man's devices had failed, by the humblest things that God, in his wisdom, has put upon this earth’ [9].

Bacterial epidemic is a continually present, if not over-used, theme in science fiction from the turn of the century, particularly with regard to the possibility for biological warfare.^4^ However, the genre remains largely silent in the wake of the twentieth century's epochal pandemic: Spanish flu (1918–1920). While unusual in one respect, this is also a reflection of a more general cultural absence on the topic with very few works of fiction responding to the flu in the inter-war period, science fiction or otherwise.^5^ Elizabeth Outka, in her critical survey of this lacuna, *Viral Modernism* (2019), refers to a ‘conspicuous literary and critical silence’, suggesting that it is precisely the scale of the devastation, combined with a general tendency to minimize representations of illness in culture, on top of the more visceral and norm-altering carnage of the First World War, which led to its relative absence [10].

By the 1940s, any reluctance towards pandemic fiction seems to have begun to fade and the theme is once more grasped by science fiction writers. In particular, the notion of a global pandemic is favoured by writers of post-apocalyptic fiction who want to ‘clear the stage’ of contemporary societies but who perhaps wish to differentiate themselves from the nuclear apocalypses which were perennial in the Cold War era. Texts such as George Stewart's *Earth Abides* (1949), Algis Budry's *Some Will Not Die* (1961) and Kate Wilhelm's *Where Late the Sweet Birds Sang* (1976) are more interested in the manner of human survival in mostly empty cities and landscapes, particularly over multiple generations, than they are in the diseases which caused such near-extinction. Stewart's novel begins with an epigraph quoting the biochemist Wendell Meredith Stanley: ‘If a killing type of virus strain should suddenly arise by mutation […]. It could, because of the rapid transportation in which we indulge nowadays, be carried to the far corners of the earth and cause the deaths of millions of people’ [11].^6^ While the protagonist and narrator Isherwood Williams is somewhat grating in his brashly confident assertions about how a new society should be reformed from the small band of survivors he accumulates and persistently condescends to, the novel does capture something of the fear of sickness and some of the consequences of a major pandemic (albeit writ extraordinarily large) such as the increased danger of secondary infections. Such details could, perhaps, be attributed to Stewart's style as a factual writer (while *Earth Abides* is firmly a work of science fiction, much of his other writing is far more realist in its approach), but it could equally stem from the author's own brush with Spanish flu which he contracted shortly after enlisting in the US Army towards the end of the First World War. Donald Scott, Stewart's biographer, notes that ‘It nearly killed him; and it would interfere with his health for decades—eventually leading him to have one lung removed’ [12]. However, given this personal detail, it is striking how clean the apocalypse is in Stewart's novel: the protagonist goes into the mountains for a holiday and when he returns, the streets are empty and quiet except for a stray dog or an aloof cat. As Rob Latham notes, this quick clean transition to an ‘after’ is ‘the chief weakness of the novel […] Too neatly, I think, and much too swiftly to be plausible. […] it is not credible that a virus, even one of “unparalleled rapidity of spread, and fatality”, could make millions of people disappear virtually overnight’ [13].

Not all science fiction diseases are apocalyptic in scale. In Frank G Slaughter's 1961 novel *Epidemic!*, rats spread plague to Manhattan: the case is identified early by an epidemiologist from the World Health Organization, but his work is not enough to stop the island needing to be completely quarantined, but it does prevent it from spreading more widely through New York and the rest of the USA. Nonetheless, the scientist's work is made more dangerous by the actions of Communist agents working against him in the city. One reason for the success and continued popularity of epidemic and pandemic fiction in the USA may be that writing about disease becomes an easy metaphor for a fear of the external threat, of something different from outside. ‘The virus’, as Anne-Marie Thomas writes, ‘is the ultimate Other in this era of globalization; it has no voice and cannot represent itself, making it a convenient enemy. And when we pit ourselves against it, the battle is often depicted as that between the individual and the collective’ [14]. Thus, in the same way that alien invasion narratives expressed fears of Communist Russia and of the ‘yellow peril’, so too were fictions which depicted disease spreading across America or the world capable of conveying a fear that American Democracy might become ‘infected’ by Soviet Communism. Conversely, the language of disease was often used to describe Communism itself: the Soviet Union ‘infected by germs of a creeping disease’ [15].^7^

Slaughter's novel is what we can describe as a ‘technothriller’, a fiction set in the present or very near future which has a science fictional core premise (a city being quarantined by disease, a rogue nuclear submarine with stealth technology, a terrorist hijacking an orbiting laser weapon^8^), but which is otherwise set in a non-science fictional or mundane world. One of the best-known writers of this type of novel was Michael Crichton, and his *The Andromeda Strain* (1969) and its 1971 filmic adaptation brought science fictional epidemics to a new level of popularity. In Crichton's novel, the disease is of extra-terrestrial origin and taps into popular anxieties about what contaminants the Apollo astronauts might bring back from their moonwalks.^9^ Deadly diseases from outside of Earth's biosphere are found recurring in science fiction, from Nicola Griffith's *Ammonite* (1992) to Ann Colbert's *The Space Between Stars* (2017). A particularly effective use of the trope comes in Kim Stanley Robinson's *Aurora* (2015), in which a crew of humans on a generation ship finally arrive on the planet their parents and grandparents set out for a century ago, only to find themselves unable to adapt to the new world or change the new world to better suit them. A mysterious disease ravages the fledgling community seemingly caused by a novel folded protein, or prion.

However, far more common is the fear of a genetically modified disease being released either in an act of bioterrorism, war or government/military negligence. As already shown, such fictions are not themselves new: Wells and others theorized the potential horrors of biological warfare in the late nineteenth/early twentieth century, but they were given new energy as the century progressed with fears of devastating disease rising alongside our understanding of how pathogenic diseases operate. Stephen King's doorstop novel *The Stand* (1978) is one such example: a weaponized strain of influenza with a 99.4% fatality rate escapes from a secret Department of Defense facility in California, almost causing the extinction of humanity: ‘Under the California desert and subsidized by the taxpayers' money’, he writes, ‘someone had finally invented a chain letter that really worked. A very lethal chain letter’ [16]. While the reference to a chain letter is now quite dated, the emphasis on transmissibility feels as relevant as ever. So much so, in fact, that King felt the need to take to Twitter to reassure his more hyperbolic followers that COVID-19 was not at all like the super-flu of his novel, ‘It's not anywhere near as serious. It's eminently survivable. Keep calm and take precautions’.^10^

Given the popularity of writers like King, it is perhaps no surprise that a book such as *The Stand* would recapture imaginations at a time of a real global pandemic. It is depressing, however, that narratives of escaped bioweapons could be taken from clearly fictional novels and proposed, without any evidence, as explanations for the current crisis. It is difficult not to attribute this to the aforementioned backlist success of fellow horror grandee Dean Koontz's *The Eyes of Darkness* (1981), where the disease ‘Wuhan-4000’ is described as ‘China's most important and dangerous new biological weapon in a decade’ [17]. Donald Trump's government did not shy from making baseless accusations which mirrored such fictions, but again it should be emphasized that though biosecurity is an issue which deserves our scrutiny and vigilance, this is the stuff of science fiction and not of our lived reality.^11^ But then, with fears of a Chinese bioweapon in *The Eyes of Darkness*, and the USA intentionally infecting the Soviet Union, China and others with the super-flu so that they take them down with them in *The Stand*, both Koontz and King are subscribing to the aforementioned Cold War approach to disease fiction, a politics rooted in distrust, fear and American exceptionalism, and so it is perhaps not surprising that Trumpian politics would come to drink from the same well.

While the Cold War ending diminished one source of pandemic metaphor fear, the events of 11 September 2001 only stoked them further and as with concerns about Communism, the threat of international terrorism was framed in the language of pandemic. For example, these remarks by Richard N. Haass, then-director of policy planning in the US State Department, to the Council of Foreign Relations in October 2001:

Sometimes dormant, sometimes virulent, it is always present in some form. Like a virus, international terrorism respects no boundaries—moving from country to country, exploiting globalized commerce and communication to spread. It can be particularly malevolent when it can find a supportive host. We therefore need to take appropriate prophylactic measures at home and abroad to prevent terrorism from multiplying and check it from infecting our societies and damaging our lives […] We also need to make sure that the virus does not mutate into something even more deadly through the acquisition of nuclear, biological, or chemical weapons of mass destruction [18,19].^12^

After 9/11 terrorism-related outbreaks of deadly diseases occur in sources as varied as Danny Boyle's reimagining of zombies as sprinting rabid forces of nature in *28 Days Later* (2002), Season 3 of real-time technothriller *24* (2003–2004), the mini-series *Covert One: The Hades Factor* (2006), Margaret Atwood's novel *Oryx and Crake* (2003), through to Dan Brown's bestselling *Inferno* (2013) and beyond. According to Dahlia Schweitzer in her study of outbreak narratives, such examples reflect a renewed sense of patriotism-driven fear during the ‘War on Terror’. She remarks that ‘while the typical outbreak narrative may function as a metaphor for modernity, the terrorism outbreak narrative literalizes the martial metaphors often used to describe disease, as well as the disease metaphors use to describe terrorism’ [20].

Lethal outbreaks of disease and all of the attendant societal changes they bring have always been used as metaphorical tools for authors to consider other topics. Looking further back, Albert Camus' *The Plague* (‘La Peste’, 1947; trans. 1948) is a particularly famous example, the plague which sweeps through the then French-Algerian city of Oran often being read as an allegory for French occupation by Nazi Germany and the author's time in the Resistance. In psychologist and author Alice Sheldon's short story, ‘The Screwfly Solution’ (1977), an infection drives men to murder women *en masse*. Rather than confront the disease, various social movements (including a new religion) pivot to try and explain the events with misogynistic rationalizations. Sheldon, who published the story under the *nom-de-plume* ‘Raccoona Sheldon’, is more commonly known by her authorly pseudonym James Tiptree Jr. and is always fascinated by gender roles and in this instance uses a disease to explore the issue. In Octavia Butler's short story ‘Speech Sounds’ (1983), a strange disease leaves society in tatters after it removes people's abilities to read, write and in some cases to speak. New languages of physical symbols are adopted by some: the protagonist's name is Rye and so she wears a pin shaped like a sheaf of wheat to represent her name. Butler's story is not about disease or sickness (although much of her work is), but it uses a pandemic to write about survival, and to cast new light on how we communicate and how slender the thread is that separates us from animals. In Nobel Prize-winner José Saramago's *Blindness* (‘Ensaio sobre a cegueira’, 1995; trans. 1997), almost everyone in an unnamed city loses their sight, causing panic and repressive attempts at containment. For Saramago, the disease is not the real focus, which is why we receive no details of how such a radical symptom occurs unannounced, or how it is spread. Rather it allows the writer to explore themes which are familiar in his work but which can now be addressed through a new lens (although perhaps the visual metaphor is not the best choice): social cohesion and the obligations of the individual to their community. Speaking of the book, Saramago focused on these elements rather than the disease explicitly: ‘I don't see the veneer of civilisation, but society as it is. With hunger, war, exploitation, we're already in hell. With the collective catastrophe of total blindness, everything surfaces—positive and negative. It's a portrait of how we are […] who has the power and who doesn't; who controls the food supply and exploits the rest’ [21]. But as we have now learned, these issues are indeed pushed to the fore under the stresses of a situation like a pandemic. In *The Years of Rice and Salt* (2002), Kim Stanley Robinson uses alternative history to suppose a scenario where the Black Death is even more destructive than it was, effectively emptying out Europe and leaving the survivors ripe for conquest. Rather than becoming a colonial force, Europe becomes a backwater. The novel spans centuries of characters up to the twenty-first century by which time the world superpowers are an alliance of Muslim states and China, with other civilizations such as the Incas pursuing their own courses. An expansive critique of colonialism and Western-centric worldviews, the novel uses a fictionalization of a real pandemic to, as Chris Pak writes, ‘imagine how a traumatic biological event that refigures the environments made available to Earth's remaining civilizations might lead to an alternate configuration of power and geopolitics’ [22].

Many science fiction novels also take real outbreaks of disease as their inspiration, with any allegorical intention a secondary concern, continuing the tradition begun by Shelley.^13^ For example, *Journals of the Plague Years* (1988) by Norman Spinrad, riffing its title off Daniel Defoe's classic account of the bubonic plague *Journal of the Plague Year* (1665), expresses Spinrad's fears that American corporate interests will stand in the way of serious progress on a cure for human immunodeficiency virus (HIV) infection. Written in the midst of the ‘AIDS crisis’, the novel is a dystopian look into a future where San Francisco is one large quarantine zone for an unnamed sexually transmitted disease and the rest of America is patrolled by ‘Sex Police’ although this is unable to contain the virus which mutates when confronted with medications or vaccines and spreads globally, ‘for twenty years, Africa and most of Asia and Latin America were quarantined by the armed forces of the America, Europe, Japan, and the Soviet Union. […] Small wonder that the Plague Years were years of madness’ [23]. Meanwhile, in the cinema, the gore-laden science fiction-horror movies of the 1980s are further examples of texts which can be read as responses to the AIDS epidemic and fears of contamination, as Edward Guerrero and others have noted of films such as David Cronenberg's *The Thing* (1982) and *The Fly* (1986), and Tobe Hooper's *Life Force* (1985) [24].

More recently, the SARS outbreak of 2003 and the subsequent H1N1 ‘Swine Flu’ (2009), Ebola (2014) and Zika (2015–2016) outbreaks have inspired more recent pandemic fictions, which in the wake of COVID have received varying exclamations of prescience from Internet sources and news outlets despite their contemporaneous reference points. For example, *Zone One* (2011) by Colson Whitehead draws on both a response to the 9/11 attacks on New York and SARS to create a literary zombie novel, which reads particularly eerily now with its populist truth-bending government of survivors singularly focused on building a wall and presiding over vast inequality of resources and safety for the survivors who are left to govern. Perhaps the most publicized of these recent texts however is Emily St John Mandel's *Station Eleven* (2014), a novel which straddled the science fiction–literary divide when it was published. A finalist for multiple literary awards including the National Book Award in the USA and the Bailey's Women's Prize for Fiction in the UK, it won the Arthur C. Clarke Award—the most prestigious science fiction book prize in the UK. I interviewed Mandel on her publicity tour in 2015 and there was no question of the book's roots, but the focus of interest for myself and many other readers was in the humanized response to extreme strain of ‘Georgia Flu’ which decimates the global human population. Among the novel's various plot threads, the most vibrant is the story of a troupe of performers a decade after the collapse of society, who make a living circling the Great Lakes putting on performances of Shakespeare plays for the small communities of survivors who cling to the shores of the lake. As Andrew M. Butler, chair of the Clarke Award judges and senior lecturer at Canterbury Christ Church University, wrote ‘while many post-apocalypse novels focus on the survival of humanity, *Station Eleven* focuses instead on the survival of our culture, with the novel becoming an elegy for the hyper-globalised present’ [25].^14^ With much of the focus at the time falling on the post-apocalyptic post-pandemic aftermath, what we neglected to note was how well the novel captured the sense of caution-fuelled paranoia that plays on the mind during an outbreak, such as when one character, Jeevan, rides a bus: ‘he found himself glancing at the other passengers. The young woman with groceries, the man in the business suit playing a game on his cell phone, the elderly couple conversing quietly in Hindi. Had any of them come from the airport? He was aware of all of them breathing around him. […] Jeevan banged the palm of his hand on the door's glass pane. Who had touched the door before him?’ [26]. In another moment which felt particularly relatable upon re-reading in 2020, the same character makes several trips to a supermarket so that he can stockpile supplies; the rest of the city are not yet taking it as seriously as he is so there is still plenty to buy: his first shopping cart is food and drink and ‘the next cart was all toilet paper’ [26].

Georgia Flu decimates the global population in a matter of days, killing 99% with an alacrity that feels more akin to the blast of a nuclear bomb than to the slow but steepening curves we are now familiar with. This more gradual approach to apocalypse is captured wonderfully in Ling Ma's *Severance* (2018), in which a disease called ‘Shen Fever’ (because it is first recorded in Shenzhen, China) spreads around the world. The fever is not lethal, but it reduces the infected to a near zombie-like state in which they mindlessly repeat their mundane routines: getting dressed and undressed, setting and unsetting the dining table, and so on without pause. In Ma's novel, the *New York Times* diligently keeps a record of the numbers of infected, New York Fashion Week carries on but with models wearing ‘face masks, gloves, and even scrubs, many branded with designer logos' and workers are encouraged to wear respirators to work while travel bans are imposed and cultural centres go into shutdown. Written as much as a satirical take on the incessant repetitiveness of modern existence, and both the mindless habits we form ourselves (particularly those enabled by the creep of technology into every aspect of our lives) and those imposed on us by our systems of work, *Severance* now reads as an uncanny foreshadowing of life in 2020 and 2021.

Another book that felt particularly prescient was Sarah Pinsker's *A Song for a New Day*. Published in Autumn 2019, the book was fresh on the shelves when the first cases of COVID-19 were being recorded in China. In an interview on the novel's anticipatory qualities, Pinsker complains ‘I was kind of looking forward to getting the story out of my head, and now we're living in it’ [27]. The narrative is set in the near future after terrorist attacks and a deadly pandemic have completely altered society: few people meet face-to-face if it can be avoided, everything is ordered online and delivered by drones, all public gatherings have been banned and social gatherings and entertainments are all now virtual online affairs. Focusing particularly on the effects of this cultural shift on the music industry (Pinsker herself is a musician as well as a writer), the book is full of phrases which we also now find in our own vocabularies such as ‘the new normal’, and calculations of safety versus freedom or happiness that are strikingly familiar:

We all felt our world slipping away, in cascades and cataracts, the promises of temporary change becoming less and less temporary. Didn't we all feel much safer? Weren't safe and healthy worth more to us than large weddings and overcrowded schools? Hadn't the pox been spread by people working and attending school when they should have stayed home? Never mind that they didn't stay home because they couldn't afford to. The talking heads were in agreement that necessity would fuel invention. Good things were coming fast, the promised; I stopped watching the news [28].

With its thoughtful analyses of what we are willing to give up to feel safe and, crucially, for how long, Pinsker's novel exemplifies the philosophical testing ground of good science fiction.

In the cinematic world, *Contagion* (2011), directed by Steven Soderbergh and written by Scott Z. Burns, and which I would categorize as science fiction in the loosest sense, was similarly returned to with new eyes and praised for its foresight. As mentioned at the beginning of this article, the film has had a particularly startling second wave of success. Unlike some of its cinematic predecessors such as *Outbreak* (1994), *12 Monkeys* (1995) or *28 Days Later* (2002), *Contagion*'s virus (MEV-1, modelled on the Nipah virus) does not spread as a result of a military experiment, terrorist attack or medical accident—rather it passes along a chain of transmission from a mundane patient zero along airline routes and familiar vectors. One scene, previously less impactful, now strikes particularly powerfully: a research scientist Ian Sussman (played by Elliott Gould) takes a break from his work on identifying the virus, and as he sits in the restaurant, he suddenly becomes aware of the people around him. Through some clever close-ups and reaction shots, Soderbergh uses his cinematography to make previously mundane actions such as stirring a hot drink, talking to a friend and touching a door handle, suddenly appear frightening and dangerous. As the epidemiologist W. Ian Lipkin, who was a scientific advisor to the production, and on whom Sussman's character is loosely modelled, states, ‘It is ironic. The reason we made this film was in fact to prevent something like this happening’ [29]. Writing on the film's original release, another epidemiologist, Larry Madoff, largely praises the scientific accuracy of the film and the realism with which it depicts the processes of the Centers for Disease Control and Prevention and the World Health Organization, but he is not universal in his admiration: ‘a disappointment to me was that the lone representative of the unofficial section, Jude Law's blogger (Alan Krumwiede) though at times ambiguous, turns out to be a nefarious opportunist’ [30]. Madoff's objection comes from the good work independent organizations can do: ‘I direct an organization, the Program for Monitoring Emerging Diseases (ProMED), […] our efforts led to the early recognition of the SARS outbreak, ahead of official notifications’. In 2020, however, Krumwiede feels like the most prescient part of the film: an opportunist Internet personality who peddles conspiracy theories and hoax remedies. He shares nothing with the reputable independents Madoff originally aligned him with, except his publishing medium. Krumwiede sows confusion and distrust of official sources: at one point fights break out over a remedy, ‘forsythia’, which he claims is a cure for the terrifying virus. Screenshots relating to this plotline were circulated on social media in China in the early days of the pandemic ‘as a coded way to express anger at the Chinese government for pushing traditional medicine remedies that had no proven benefits’ [31]. But in time, this plotline would also have familiarities in the West with resonances in the discourse of alt-right Web outlets and so-called anti maskers, and in the claims for the remedial benefits of hydroxychloroquine. Conversely, UK Health Minister Matt Hancock credited the film with improving his appreciation of the challenges of a national vaccination programme: ‘I wouldn't say that film is my primary source of advice on this' he reassured, but added ‘In the film it shows that the moment of highest stress around the vaccine programme is not in fact before it's rolled out, when actually it's the scientists and the manufacturers working together at pace, it's afterwards when there is a huge row about order of priority’ [32]. Overall, *Contagion* was an effective (and affective) film even before COVID-19, its realism adding an unexpected new layer to Hollywood's previous offerings which enhance the scariness of the prospects it presents. However, through successful storytelling, the filmmakers are able to manage that fear. As Jane Doherty and James Giordano write, *Contagion* ‘get[s] audiences to question the source of fear, and recognize the danger of misinformation’. ‘Fiction’, they continue, ‘enables us to examine our fears and sources of information about COVID-19’ [33].

This notion from Doherty and Giordano of fiction as analytical is key. Science fiction is not prophetic: for every accurate detail in a work, you can find a plethora of ideas which did not come to pass. Science fiction cannot predict where the next pandemic will come from, or what variety of pathogen will be behind it. However, science fiction can anticipate how we as individual humans and our societies will continue to change, adapt and respond to the new situations with which we are confronted. The COVID-19 pandemic has already caused a huge shift in the way we interact with others, the way we think about ourselves as individuals and the ways governments think about their responsibilities. The extent to which these changes will prove lasting, and if not how quickly they will begin to fade from view, remains to be seen, but science fiction has always been interested in these issues and will continue to provide a thought experiment laboratory of infinite scale in which to examine them. As science fiction author and English literature professor Adam Roberts notes in his study of apocalyptic fictions, ‘it's too early to say how coronavirus will factor into our ongoing general fascination with the end of the world. But it illustrated a core truth about human beings: we *are* our interactions with others’ [34]. By the time something like normality returns to everyday life, it could be that so many people want to get on with their lives and put the pandemic behind them that realist, so-called non-genre, fiction struggles to find a market and a voice with which to tell stories about this moment in our histories, much as it did after Spanish flu.

However, with the added distancing effect of its tropes and plot devices, genre fiction seems far more equipped to place our experiences into a framework which we understand, whether thriller, romance, horror, fantasy or crime novel. It is difficult to disagree with Costello *et al*. in their call to action, ‘for other historians of futures past to help uncover timelines, and write alternative fictions, that promote pedagogies of hope, care, justice, and a brighter day’ [35]. This comes as part of a piece which creates a new work of science fiction as a device to look back on and reflect upon our own times and I certainly hope such texts emerge. Before they can, however, I suspect we will see a period of coming-to-terms, an attempt to reframe ourselves and our world, an attempt to understand. By engaging with the issue of pandemics through the lens of cognitive estrangement, science fiction is able to both grasp and escape from the issues raised by our COVID-19 responses. It can bring plausibility to the most unbelievable elements of our experiences while making use of metaphorical and allegorical devices to interrogate the implications and potential consequences of our familiarity with others. Science fiction seems set to continue its more-than-200-year tradition of giving us new ways to think, new ways to approach our past and our future, and new ways to learn.

## Endnotes

1 Laurel Bollinger has written about the science fiction of microbiologist Joan Slonczewski and others which explores a symbiotic relationship with viruses, reflecting research which shows the presence of viral DNA as part of the human genome. These are generally more positive tales of immunology and virology than the texts explored in this paper. See [5].

2 Percy Shelley (1792–1822) and Lord Byron (1788–1824) are represented in the novel by thinly fictionalized representations: as Adrian and Lord Raymond, respectively.

3 Helena Costa and Josep-E Baños provide a detailed outline of both the scientific and social contexts of Wells' story, which may well be the first example of bioterrorism in fiction, in their article [8].

4 Alongside Wells, a notable writer on this theme is the American writer Jack London (best known for his adventure novels *The Call of the Wild* and *White Fang*). London returns to the idea on numerous occasions including in a future war in the racist short story ‘The Unparalleled Invasion’ (1910) and the post-apocalyptic novel *The Scarlet Plague* (1912).

5 Of those that do exist, the best known is *Pale Horse Pale Rider* (1939) by Katherine Anne Porter who survived the illness.

6 Although he does not give a full citation, Stewart is correctly quoting Stanley's article [11].

7 American diplomat and historian George F. Kennan, quoted in [15].

8 Latterly, the premises of Tom Clancy's *The Hunt for Red October* (1984) and the James Bond film *Goldeneye* (1995), two preeminent examples of the technothriller genre.

9 While not as explicitly anti-science as many of his later works such as *Jurassic Park* (1990) and *State of Fear* (2004), it is worth noting that the scientists are not the ones who save the day in the novel, and indeed removing them from the book entirely would not alter the ultimate conclusion.

10 8 March 2020: https://twitter.com/StephenKing/status/1236782826911150080.

11 In just one example of this sort of misinformation, Secretary of State Mike Pompeo remarked in an interview that ‘I can tell you that there is a significant amount of evidence that this came from that laboratory in Wuhan’, *ABC News This Week*, 3 May 2020 (https://abcnews.go.com/ThisWeek/video/secretary-state-mike-pompeo-70478299).

12 Quoted in [18,19].

13 Indeed, Camus too drew on real instances of disease in Oran when writing *The Plague*. The novel is thought to be based on research he did into the 1849 cholera outbreak in that city.

14 Andrew M. Butler, quoted in [25].

## Data accessibility

This article has no additional data.
